# A stakeholder co-design approach to designing a dental service for adults experiencing homelessness

**DOI:** 10.3389/froh.2024.1355429

**Published:** 2024-08-26

**Authors:** Declan Cairns, Andrea Rodriguez

**Affiliations:** School of Dentistry, University of Dundee, Dundee, United Kingdom

**Keywords:** co-design, dental services, homelessness, inequalities, qualitative research, participatory research, dental services for adults experiencing homelessness

## Abstract

**Introduction:**

The homeless population faces a “cliff edge of inequality” when trying to access essential dental services. There are several additional barriers to accessing dental care in comparison to the general population and the heterogeneous nature of patients presents a significant challenge when designing dental services to meet their needs. Following the Smile4Life report in 2009, there is limited up-to-date and population-specific evidence available for the optimal model of service delivery.

**Aim:**

This study aimed to co-design principles for a prospective dental service for adults experiencing homelessness.

**Methods:**

A qualitative methodology was used to incorporate experts-by-experience into elements of co-designing a dental service for adults experiencing homelessness. The study combined elements of an experienced-based co-design framework for healthcare innovation with community-based participatory research. Focus groups with people experiencing homelessness and healthcare practitioners were conducted to identify principles for any prospective dental service, as well as several barriers and enablers to establishing a homeless dental service. The findings were transcribed and analysed using thematic analysis on Nvivo software.

**Results:**

From the qualitative analysis five key themes emerged: (1) Impact and expectations of oral health while experiencing homelessness, (2) Barriers to accessing dental care; (3) Practitioner's views about homelessness and access to care; (4) Barriers to designing a homeless service and (5) Enablers for co-designing a new model of dental care delivery for the homeless population.

Five key principles for a new model of homeless dental service were identified: (i) Services designed to address the needs of patients; (ii) Services delivered in a safe and welcoming environment (iii) Training and consistency of staff; (iv) Focus on dental education (v) Developing peer mentoring and peer support.

**Conclusion:**

While the barriers to accessing dental care while homeless are well established and understood by healthcare practitioners, more work is required to gain consensus on the most effective way to deliver an innovative a sustainable dental service for patients experiencing homelessness. Previous negative experiences, lack of readily available information on services and barriers to access in the current system could be addressed by developing peer mentors within the homeless community, empowering individuals to share their knowledge and skills to support others in improving their oral health.

## 1 Introduction

Homelessness is not only a profound form of social exclusion but also a pressing global public health concern. Social, economic, cultural and political factors affect the extent and severity of an individual's experience of this social exclusion (1). It is the intersectionality of multiple disadvantages (2), combined with poverty (3), that often leads to an individual becoming homeless. Because health is considered to exist on a gradient (4), Freeman argues that individuals at the lower end of this scale are more acutely affected by the ‘causes of the causes’ of ill health (2). These vulnerable groups are also affected by the inequitable distribution of health resources, identified by the inverse care law, which that recognises individuals with the highest level of health needs in a society, often face the greatest barriers to accessing appropriate care (5).

In particular, the homeless population have a greater experience of ill health in comparison to the general population (6), but also faces additional psychosocial barriers to accessing healthcare (7). These additional barriers often drive patients, who are already vulnerable, to disengage from mainstream health services and this lack of opportunity for preventative advice and treatment increases the incidence of comorbidities (8) and leaves patients facing a “cliff edge of inequality” (2). Aldridge described the extent of their ill health experience as “extreme health” (8) and Freeman et al. built on this concept to suggest the same social determinants of health affect an individual's oral health and labelled this phenomenon “Extreme oral health” (2).

Patients experiencing homelessness generally have; higher levels of dental anxiety than the general population (7), higher levels of dental disease requiring treatment (7), increased missed appointments due to their chaotic lifestyle and are more likely to have been stigmatized for the presentation of their teeth and mouth (9). This frequently leads to patients only attending emergency dental appointments when in pain (10) which can be challenging for general dental practitioners to accommodate within the current model for delivering services. The challenges of delivering effective dental care for this population are well established in the literature, however, because of the heterogeneous needs of the homeless population's oral health (11), there is no “one size fits all” approach to developing effective services.

Freeman suggested the adoption of co-design to empower vulnerable groups facing social exclusion would lead to developing interventions that understand and remove the exclusory elements that underpin many services for people experiencing homelessness (2). Participatory research (12) helps policymakers to understand the views and experiences of individuals experiencing homelessness and incorporate them throughout the design and implementation stages of service development (13).

In Scotland, 32,242 households were assessed as homeless in 2022–23 (14). The true number is thought to be much higher, as these statistics don't account for the “hidden homeless” population that is staying in temporary accommodation or sofa surfing. It is important to build a robust evidence base of solutions to improve the oral health of this vulnerable population, tailored to their specific needs. Therefore, this study aims to co-design an innovative, sustainable dental service for adults experiencing homelessness.

## 2 Methodology

A qualitative methodology was used to incorporate experts-by-experience into elements of co-designing a dental service for adults experiencing homelessness. In this context, experts by experience were defined as “someone who can articulate lessons and suggestions from their own ‘lived’ experience of homelessness and health care challenges (15). The study combined elements of experienced-based co-design framework for healthcare innovation (16) with community-based participatory research (17). This was done to identify the key principles, barriers and enablers to establishing a dental service from the perspective of people experiencing homelessness and healthcare practitioners. This research followed, and adapted, the participatory and multi-disciplinary co-design framework (16) composed of three stages: Pre-design, Co-design, and Post-design (Table 1).

**Table 1:** Seven steps in the co-design framework for healthcare innovation.

Pre-design	Co-design	Post-design
**Step 1—Contextual inquiry** - Participant observation and patient public involvement was conducted in soup kitchens, homelessness services and on the streets that surrounded the area where key homelessness services were based - Understanding the oral health needs and expectations of individuals experiencing homelessness to inform discussion in the focus groups - Discussing the research question with managers and practitioners in health Boards and third sector to inform discussion in the focus groups **Step 2—Preparation/Planning for participation** - Engagement with participants from both groups (people with lived experience of homelessness and health care practitioners). - Informal Patient and Public Involvement (PPI) sessions to frame the issue and inform - Selection of a facilitator - Providing participant information sheets to both groups - Sourcing materials to support data collection (recording device/location…) - Testing the instruments and making necessary change	**Step 3—Framing the Issue** - Focus groups (Group 1 and 2). - Presentation of generative design work. - Participants and facilitator engaged in critical reflection of their experiences - Appreciative inquiry—highlighting the positive aspects of attending a dental clinic to inform **Step 4—Generative Design Ideas** - Identification of oral health needs and expectations about accessing dental services - Knowledge exchange about actual experiences attending dental services while experiencing homelessness - Identification of challenges in accessing dental services - Starting a process of re-imagining/re-creating a new model of dental service provision **Step 5—Sharing Ideas** - Identifying principles to base the foundation of this new model - Discussing what must change in actual services - Consolidation of the process of re-imagining/re-creating a new model of service delivery - Shared vision for the future	**Step 6—Data Analysis** - Preparation (transcription, analysis framework) - Organizing (coding) - Reporting findings **Step 7—Requirement translation** - Combination of first and second focus group to identify key themes for service development - Identify feasible priorities to consider - Action items identified 43

Adapted from M Bird (16).

Experience-based co-design and participatory research can be valuable tools to address the power imbalances (18, 19) between marginalised groups and those in positions of authority when designing services. This type of research empowers community members to identify their needs and work collaboratively with researchers to facilitate any changes to policy or practices as required (16).

### 2.1 Participant recruitment

Research participants were recruited by the principal investigator (DC) and divided into two groups. Group 1 was formed by six people experiencing homelessness called the “experts by experience”. Group 2 was formed by six practitioners involved in developing or delivering oral health promotion and dental services.

Regarding group 1, a purposive sample of people experiencing homelessness was formed through activities delivered in partnership with two NGOs. Informal patient and public involvement (PPI) sessions were organised at a weekly soup kitchen, where members of the community were invited to participate in research and share their views on accessing dental care while experiencing homelessness. Following guidance from the National Institute for Health and Social Care Research (20) participants received compensation for their participation, in the form of £30 shopping vouchers. However, participants were not aware of the voucher, or its value, before accepting to take part in the focus group. Participants were also made aware that they could withdraw at any time of the research process. The second group of participants was recruited through the network of the principal investigator. Recruitment emails were sent to practitioners working within the National Health Service (NHS) linked with national homelessness programmes. This second group was comprised of General Dental Practitioners, Oral Health Educators; NHS Managers; and a Dental Public Health Specialist.

### 2.2 Seven steps in the co-design framework

In Table 1, the seven steps in the co-design framework for healthcare innovation, adapted from M. Bird (16), were presented.

#### 2.2.1 Step 1: contextual inquiry

Step 1—The contextual inquiry for this research was based on both researchers’ experience working with adults experiencing homelessness in Scotland. The principal investigator (DC) had previously led work to establish a charity organisation focusing on oral health improvement for the homeless community, and for many years worked directly with local soup kitchens to deliver dental supplies and dental advice. In addition, both researchers (DC; AR) have been actively involved in previous national and local health needs assessments of this population (7) and in the development of a service mapping framework (21) to inform policy and service design. Some of the key challenges identified through these needs assessments prompted this research question. The importance of involving and listening to the voices of socially excluded groups facing homelessness regarding the most effective model of delivering dental care is a key principle of this study.

In Step 1 participant observation was conducted in homelessness services. Informal patient and public involvement sessions (PPI) were carried out to engage with adults experiencing homelessness, in order to assess their willingness to share their experiences accessing dental care while homeless. Individuals who were happy to share their knowledge were invited to attend a well-established community hub for adults experiencing homelessness, where the first focus group was delivered. Participants from this first focus group were also invited to attend the second focus group. Healthcare practitioners in group 2 were recruited within NHS boards.

#### 2.2.2 Step 2: preparation and training

In Step 2, the principal investigator acted as facilitator for the conduction of both focus groups. The facilitator had extensive experience working with service users and members of senior management within the NHS boards and third-sector organisations. The role of the facilitator was to contribute to the conduction of the focus groups, tailoring the communication style for each group accordingly, to make participants feel as comfortable as possible in sharing their experiences and knowledge. The facilitator used Tables 2, 3 as a guide to facilitate conversation among participants.

**Table 2:** Themes, descriptive questions and prompts for workshop 1 (experts-by-experience with lived experience of homelessness).

Themes/topics to be explored—workshop 1	Broad descriptive questions
Knowledge/Awareness/Needs of Participants in Relation to Oral Health	What do you understand is having a healthy mouth? What behaviours or habits do you have that contribute to your oral health? (Good and bad) Do you go to the dentist for check-up or only when in pain?
Experiences trying to access a dentist in the last 18/24 months…	Describe your experiences trying to access the dentist? *(Positive and Negative experiences)* How did those experiences make you feel?
Reactions/aspirations to different models of dental care used throughout the rest of the UK…	Current model—Attend general practice? Supported to attend general practice? Designated service—Hospital/Community location? Emergency treatment only?

**Table 3:** Themes, descriptive questions and prompts for workshop 2 (experts-by-experience with lived experience of homelessness and healthcare practitioners).

Themes (topics to be explored)—workshop 2	Broad descriptive questions
Needs and contexts of people experiencing homelessness	Introduce previous discussions from the first focus group around barriers and enablers… Consider current service provision…In what ways does it meet the identified needs of patients?
Service components	What should a high quality dental service for adults experiencing homelessness look like? In an ideal world (blue sky thinking): What should the core values of the service be? What difficulties might arise trying to set up a service? Realistic roadblocks to overcome?
Service design and healthcare system integration	Given topics discussed today and expertise of participants, detail options for a prospective dental service for adults experiencing homelessness. Relative to your field of expertise, how feasible are these options? Can this dental service be linked into other homeless services?

The third-sector organisation, Simon Community Scotland, helped to establish the optimal approach to engaging with patients experiencing homelessness through their extensive experience working with the community homelessness and by offering the use of space in their multi-functional Hub. The Hub is a psychologically informed environment (22) for adults experiencing homelessness that is designed to be an inclusive space that makes individuals feel safe and comfortable.

Both focus groups were held in the Simon Community Hub. An audio recording device was used to record participants’ responses, which was tested in the location before each workshop. Participants were provided with a participant information sheet in advance of the focus group and the contents of this and the consent form were read aloud to compensate for any reading or writing comprehension issues. Participants in both groups were asked to sign a consent form before starting. Support was offered for any participant requiring assistance to better understand the research process.

### 2.3 Co-design

#### 2.3.1 Step 3–Step 5

The co-design process involved Step 3: Framing the issue; Step 4: Generative design ideas and 5: Sharing ideas.

Informal PPI sessions were used to gather insights from individuals with lived experience of homelessness and their recent or historic experiences accessing dental care. This information was then used to inform the broad descriptive questions and themes to be explored during the focus groups, to allow participants to expand on their experiences and generate innovative ways to overcome barriers to accessing care.

Table 2 highlights the themes explored and broad descriptive questions in the first focus group with Group 1 (experts-by-experience with lived experience of homelessness). Table 3 illustrates the themes explored and broad descriptive questions used in the second focus group with Group 2 (Experts-by-experience with lived experience of homelessness and healthcare practitioners).

Research participants engaged in critical dialogue and reflection during both focus groups and shared their views and experiences towards the conceptualisation of a model of care that would meet the needs of adults experiencing homelessness. Key themes presented in Tables 2, 3 were used to guide the discussion in each respective focus group.

Table 4 highlights key characteristics of participants in both focus groups.

**Table 4:** Participant's characteristics for both focus groups.

People with lived experience (PwLE)	Gender	
Participant 1	Male	
Participant 2	Female	
Participant 3	Male	
Participant 4	Male	
Participant 5	Female	
Participant 6	Female	
Facilitator	Male	
Health Care Provider (HCP)	Gender	Role
Participant 1	Male	General Dental Practitioner
Participant 2	Female	Public Dental Service Clinician/Clinical Service Manager
Participant 3	Male	Specialist in Dental Public Health
Participant 4	Female	Oral Health Educator/Oral Health Improvement Manager
Participant 5	Female	Operational Manager for Oral Health Improvement Manager
Participant 6	Male	Participant with Lived Experience of Homelessness
Facilitator	Male	Same Facilitator as other focus group/primary investigator

Some of the participants of Group 1 (people with lived experience of homelessness) had known the facilitator through engaging at a charity soup kitchen, which established trust and open communication between the group. Many of the participants in Group 2 had previously worked in healthcare services alongside each other, which quickly established a rapport among the group.

### 2.4 Post-design

#### 2.4.1 Data analysis

The key themes and information from the first focus group were analysed and used to inform the discussion in the second group. In an iterative process, the audio recording from the first group of participants was transcribed by a single researcher, using the 6 stages of thematic analysis identified by Braun and Clarke (23), to interpret the data. These were: data familiarisation, generating initial codes, searching for themes, reviewing potential themes, defining and naming themes, and producing the report. After the researchers (DC, AR) independently examined the data, they met together to discuss their categories and themes. When a disagreement occurred, further discussions ensured that a consensus was reached. Key themes were identified from the first focus group and used to inform the discussion of participants from the second group. This was done to give the practitioners a context to consider when discussing service design and delivery, to ensure they fully understood the multi-dimensional and relational elements of social exclusion experienced while homeless (2). The data collected from the second focus group was analysed and interpreted using the same process on Nvivo software.

#### 2.4.2 Requirements translation

Once both focus groups had been transcribed and sorted into initial codes and themes, the results from both groups were compared to identify common suggestions for a future homezless dental service. This included the principles embedded in the service provision, barriers to accessing healthcare services, the barriers to establishing any service from a practitioner perspective and the underlying principles that policymakers should consider to when designing a dental service for people experiencing homelessness.

The results from each feedback, once combined, were developed into mind maps using MindView software. This helped to give a visual representation of the key themes discussed in both groups to compare and contrast the responses given.

Ethical considerations: Ethical approval was obtained from the Research Ethics Committee at the University of Dundee (UREC number UOD-SREC-SDEN-2022–007). Participant information sheets were provided, and consent forms were required to be completed before taking part in the study. All of the data were anonymised and confidentiality was ensured.

## 3 Results

The qualitative findings are described below. Five key themes emerged from the combination of the data analysis from both groups.

The group of participants with lived experience of homelessness highlighted the first two research themes: (1) The impact and expectations of oral health while experiencing homelessness, and (2) The barriers to accessing dental care while experiencing homelessness. The group of participants composed of practitioners highlighted the following themes: (3) Practitioner's views about homelessness and access to dental care; and (4) Barriers to designing a dental service for experiencing homelessness.

Both groups of participants discussed theme 5: Principles for co-designing a new model of dental care delivery for people experiencing homelessness. The results from each group were combined and identified five key principles for establishing an inclusive new model of dental health service that can respond to the health needs and aspirations of people experiencing homelessness.

### 3.1 Focus group 1: the views of people with lived experience of homelessness

#### 3.1.1 Theme 1: impact and expectations of oral health while experiencing homelessness

The negative impact of poor oral health while experiencing homelessness was a strong theme throughout the discussion, with participants reporting experiencing stigma and judgement as a result of poor oral health. Participants reported they had faced stigma and judgement from society due to the condition of their teeth, which harmed their mental health and overall confidence—particularly when applying for jobs. One participant with lived experience of homelessness (PwLE) reported:

*“People assume that if you’ve got rotten teeth or teeth missing or brown teeth that, automatically, he's on drugs… Or he can't be trusted, or he's been begging. It's all those judgmental words that come out of people…” [PwLE participant]*

Despite facing discrimination because of the aesthetics of their teeth, participants had relatively low expectations for what constitutes a healthy mouth. Most of the participants were focused on having a mouth that was “functional” and “not in pain”, while other participants wanted to avoid embarrassment while talking with others and/or having to pause and think about the ingestion of particular foods they would like to eat.

#### 3.1.2 Theme 2: the barriers to accessing dental care

Three sub-themes on the barriers to accessing dental care were identified: (i) Chaotic life structure; (ii) Lack of Trust; and (iii) Previous bad experiences with a dentist. Knowing what these barriers are can inform policymakers and practitioners when designing inclusive and sustainable dental services to empower patients to overcome them and improve their oral health.

##### 3.1.2.1 Chaotic life structure

The most common barriers that were discussed to accessing healthcare were linked to a chaotic life structure while someone is sleeping rough or living in temporary homeless accommodation, such as experiencing or suffering violence or drug misuse.

Participants were not satisfied with temporary hostel accommodations, reporting a high prevalence of drug use, violence, and poor living conditions. This challenging environment left them feeling “*emotionally exhausted*” as they had to deal with multiple demands, and they were keen to leave the accommodation as quickly as possible—often without seeking dental care services or completing their oral hygiene routine. Another PwLE highlighted:

*“Especially when you’re staying in a hostel you just get used to getting up and out… One morning you’ll brush them and the next you won't, it's just one of those things you need to remember.” [PwLE participant]*

While staying in homeless accommodation, there was constant upheaval and often episodes of extreme violence, which was particularly traumatic for some participants and significantly impacted their mental health. Participants indicated they had become used to “*living out your bag*”, because of moving about regularly or rough sleeping, and it was difficult to maintain a regular oral hygiene routine because of this.

The abuse of substances was another factor that inhibited a healthy routine identified by participants as habits like smoking marijuana and falling asleep before brushing their teeth at night. Participants openly discussed their previous issues with substance misuse and highlighted that vulnerable drug users are often targeted in the hostel accommodations by drug dealers, which makes recovering from any dependency even more challenging in this environment.

Significantly, one participant reported that he had a positive experience being supported by a member of staff in a hostel that used a harm reduction approach, and he found this to be particularly supportive in helping him in recovery.

These challenges to engage with mainstream services continued every day after leaving the hostel, as it is common to be “scattered from the east to the west” within the homeless system—and as a result, is not unusual to have to walk long distances to access services or support.

##### 3.1.2.2 Lack of trust

Building trust with staff was challenging for participants as they reported being referred to multiple different services and service providers while experiencing homelessness. The need to constantly repeat their experiences and often retell traumatic events often led to frustration, being “*passed off from this person to that person*” within the homeless system, which led one PwLE to respond that:

*“They are not bothered about you or your outcome, you’re just another person to them. You’re just another one on the list.” [PwLE participant].*

This attitude is illustrated by the phrase “*services make problems not solve them*” which highlights the lack of trust that participants in services delivering for their needs. They identified reception staff demanding identification and proof of address, which many participants did not have, as a barrier to registering at a dental practice, and also led to them feeling judged negatively.

Most participants reported only seeking dental treatment when they experienced extreme pain, however, the majority admitted calling their GP to seek dental advice or access medication for dental pain.

The group also discussed that there was a lack of readily available information on how to access dedicated homeless services. Word of mouth was identified as a very effective way of disseminating this information, especially through a central location such as a drop-in service.

##### 3.1.2.3 Previous bad experience with a dentist

Participants detailed several negative experiences when attending a dentist, some of which had left a lasting negative impact on individuals. In particular, the approach and mannerisms used by dental professionals often came across as “*being told off*” and lectured, which they felt was “*judgmental*” and that the dentist had failed to make any effort to listen to them and understand their perspective. This was particularly evident when participants discussed dental practices issuing fines when they missed appointments, which they felt were unfair and unrealistic.

Participants reported a significant amount of anxiety around different dental procedures, with the use of the injection to numb the teeth reported as the most common. Because most participants were anxious about other factors like the noise of the drill, as well as an injection, many reported delaying seeking treatment until they could not cope with the pain. They felt that dental treatment was very invasive, and because of previous trauma and a lack of trust between the patient and the dental professional, it left one participant feeling quite vulnerable when lying back in the dental chair.

The attitude and mannerisms of dental staff left patients with a negative perception of dental care professionals, which was a significant barrier to accessing dental services for most participants.

### 3.2 The views of healthcare practitioners

#### 3.2.1 Professional views of homelessness and access to healthcare

The second focus group was composed of dental care professionals (DCPs) who shared their understanding of the barriers to accessing dental care while experiencing homelessness. The group demonstrated a very good understanding of the barriers to mainstream dental care, many of which were similar to the barriers identified by service users in the first workshop.

In general, previous negative experiences with a dentist were identified as causing high levels of anxiety among patients. While this is not unique to patients experiencing homelessness, participants in this group identified several factors combined, such as stigma and judgement coming from professionals, to make it particularly difficult for these patients to overcome this hurdle. There was consensus that patients experiencing homelessness usually delay seeking dental treatment until absolutely necessary, which often means they present for emergency appointments in extreme pain.

Participants identified that the people experiencing homelessness generally have an extremely chaotic lifestyle that can be challenging to navigate. They also identified that the needs of patients who have recently been made homeless are very different to those who have been in the system for a long time and that the current system is not tailored to address their needs. Issuing fines and deposits was acknowledged as a barrier for patients on a low income to finish dental treatments and this was also applicable when patients were asked to provide identification, despite often having no fixed address or access to benefits. Participants felt this approach needed to be changed as it can have significant negative impacts on patients trying to access basic dental care.

#### 3.2.2 Barriers to designing a dental care service

Participants gave their views on what would be the main barriers to designing and implementing a designated homeless dental service. These barriers were sub-categorised into governance, financial sustainability and the limitations of the current system.

##### 3.2.2.1 Governance

There were several issues raised about the governance of any potential homeless dental service to be created, particularly if the service is based in a community location. Seeking to learn from existing models of care for marginalized groups, the discussion focused on the challenges of using a non-NHS premise to operate NHS services—and the issues this raises about who is responsible for running the service. In particular, the maintenance of equipment and instruments was highlighted as a potential problem. The established model of a mobile dental van to provide dental treatment for vulnerable groups as people experiencing homelessness in disadvantaged areas was discussed for comparison. The existing challenges with communication between organisations and sectors were also acknowledged as a barrier to establishing any new service. Participants agreed that organisations tend to work in “silos” and currently, communication is largely between individuals and not strategically across organisations. Participants suggested these links are difficult to maintain when members of staff that have established this inter-sectoral communication, move on from the organisation—especially when different services use different operating systems.

This was a concern and needed consideration when designing a multi-disciplinary service, as effective communication between organisations was identified as essential for delivering services that meet the diverse range of needs of the patients.

##### 3.2.2.2 Financial sustainability

Sustainable staffing costs were also discussed, including the possibility of making a salaried post within the NHS that would take responsibility for operating the service. However, participants indicated that consideration would have to be given to both the initial cost of recruiting employees and the recurring “on-costs” when establishing a new service and importantly whether funding could be repeated to keep the project running in the long term.

The commissioning of a mobile dental van brought up examples from other health boards that indicate this model would potentially carry a significant up-front cost to establish, without necessarily improving the accessibility or quality of care for patients. It was suggested that funding may be available for establishing innovative projects for vulnerable groups, however, participants identified that an issue with current funding is that it is predominantly non-recurring and this poses a significant challenge to the sustainability of any service.

##### 3.2.2.3 Limitations with the current dental system

The limitations within the current dental system were also highlighted as a significant barrier to co-designing a new model of dental care service. In particular, the policy of lifelong registration with a dentist makes it difficult for patients who have moved away from a particular area to access dental treatment in a different location. Participants indicated this issue is compounded by the ongoing problems accessing mainstream NHS dental practices. Multiple participants with lived experience of homelessness (PwLE) reported in the first focus group that it was easy to book an appointment with a local dentist prior to Covid-19, however, this has changed following the pandemic. One of the health care practitioners (HCP) acknowledged the additional challenges that not exist trying to access care:

*“Because of Covid, nobody is taking on new patients. There has been a problem, which has been multiplied… You’ve got the patients trying to register and there's nowhere to put them. The situation is difficult and it's been made worse, currently, by the Covid situation.” [HCP participant]*

Participants also identified a significant overlap between individuals experiencing homelessness and other vulnerable groups—such as prisoners or patients accessing addiction services. The impact of life in prison on the oral health of individuals and the challenges to access dental care after being released. Alongside other priorities that community returners face, which do not include oral health, were discussed. As the public dental service is the mainstay for delivering care for these vulnerable patients, participants recommended strong consideration should be given to how we include these groups in any future service provision.

### 3.3 Focus group 1 and 2 combined responses on principles to co-design a new homeless dental service

The final theme emerged from both groups of participants and identified five principles that should be embedded in the co-design of a new model of dental care for people experiencing homelessness:

(i) *Services designed to address the needs of patients;*
(ii) *Services delivered in a safe and welcoming environment;*
(iii) *Training and consistency of staff;*
(iv) *Focus on dental education;*
(v) *Developing peer mentoring and peer support*.

There was significant convergence between participant's responses in the first and second focus groups around the barriers and challenges accessing dental care while experiencing homelessness. There was a similar convergence of ideas around the value of education and using peer mentors to deliver information to “hard to reach” groups. The main area of divergence of opinions was around the ideal location any prospective dental service, with HCP participants illustrating the challenges and costs associated with establishing a new service, while PwLE participants reinforced their barriers to accessing care within the current system.

#### 3.3.1 Services designed to address the needs of patients

Participants identified that any service should be tailored to be effective in addressing the needs of the homeless population: “*A service that fixes problems not makes them*” *[PwLE participant]*.

Participants with lived experience of homelessness revealed that if a designated homeless dental service could be established, and delivered ‘differently compared to a “normal” high street dentist’, they would be more likely to attend. Trust and positive relationships previously established with patients were considered essential and it was suggested that a similar approach to create an integrated homeless dental service could be a platform to engage patients with other health issues such as substance misuse.

However, the views of dental care professionals were that reaching and treating vulnerable groups such as homeless patients was the remit of the Public Dental Service. Because of this, there was a need to consider patient groups facing social exclusion and guarantee that any service is inclusive and sustainable. As the oral health needs—and priorities—of someone experiencing homelessness can change dramatically, often from day to day, any service should try to accommodate this:

*“It's almost like the need for two services, someone that has an acute problem who needs their toothache fixed. How do we deal with that acute problem, and actually when people are ready and engaged, how do we get them fit and integrate them back into general practice.” [HCP participant]*

Participants discussed this model positively, highlighting it as a way to resolve acute dental problems, while also achieving long-term engagement through building trust and rapport. The potential benefits of a “*one-stop shop*” service were discussed particularly as a way to manage patients’ acute anxieties and give them a positive dental experience:

*“That sort of concept of a one-stop shop that can solve many problems is brilliant. And that will certainly address some acute problems for individuals, regardless of what that service is. Whether it's dental or whatever…. Once you’ve got past that (initial) bit and people want to make that change” [PwLE participant]*

The second group of participants identified the potential of practitioners who already work in national programmes to support these patients in attending dental appointments by sending personalised and positive reminders and accompanying them in some sessions.

#### 3.3.2 Services delivered in a safe and welcoming environment

Building on the theme that any potential service should be designed around the needs of its patients, both groups identified the importance of an appropriate and welcoming environment to deliver care that should make patients feel safe and comfortable. One participant highlighted that this should be “*colourful*” and “*bright to the eye*” while others in the first group focussed on it being easily accessible, based in an area that they are familiar with and based in a location where they wouldn't have to travel long distances to utilise. A common theme identified by both groups was the need for any service to have an element of flexibility built into it. A healthcare practitioner who has experience working with patients experiencing homelessness reported their approach to managing their diary around the needs of patients:

“We would maybe double book them or try to put them in at a time that we knew we would have a wee bit of flexibility… Without turning someone away, because we would never really want to do that. It was just about managing things, and understanding from both ends. And that is difficult” *[HCP participant]*

#### 3.3.3 Training and consistency of staff

Participants mentioned the importance of communication style and the approach used by staff in any service as playing a significant role in improving client engagement. In particular, the first group of participants indicated they found regular staff changes very unsettling. Because many patients experiencing homelessness have faced judgement and stigmatisation accessing healthcare, they reported it was difficult to open up about previous traumatic experiences that often drive many of their anxieties and subsequent unhealthy behaviours. When they did build this relationship with a practitioner, they reported finding it frustrating having to repeatedly recount their trauma to a different professional:

*“Just say you had something that you’re insecure about. And say you’re in a dentist and I tell you that, right? But you don't put that on paper, that's just something between me and you. See two or three weeks later, it's a new guy… and he doesn't know that.”* *[PwLE participant]*

Having staff well-trained to understand the barriers to maintaining a healthy mouth while experiencing homelessness as well as more information on how to access dental services was perceived as essential to make patients feel welcomed and comfortable discussing their oral health.

Participants reported that when feeling listened to and being offered achievable solutions to their issues this helped them build positive relationships with dental care professionals. More training on trauma-informed care was perceived as a gap among dental professionals. As well as ensuring the staff recruited are empathetic and compassionate, most participants agreed that appropriate training should be delivered to all dental care professionals to make sure they understand the needs of their patients as the majority of them likely never experienced social exclusion and vulnerability to this extent:

#### 3.3.4 Focus on health education

Education was another central feature of both focus groups of participants. Experts by experience in the first group indicated they would benefit from having more access to information about improving their oral health and how to access dental services:

*“If you knew how to manage them and you learned what was bad for them and so forth. If you were to bring more knowledge, you would bring more comfort to people” [PwLE participant]*

Healthcare practitioners in the second focus group identified that Smile4Life's remit is to deliver oral health training to “*as many people as possible*” to upskill and develop key workers within homeless services. These individuals are then able to give advice and support to patients on their oral health concerns, as well as signposting effectively to available dental services.

There was convergence amongst the HCP participants in the second focus group, who championed having empathetic clinicians who understand the complex needs of their patients as a significant factor in promoting engagement. Highlighting that many healthcare practitioners have not experienced the adversity and additional barriers to accessing care faced while experiencing homelessness, one HCP participant explained:

*“Most people who work in healthcare, most people not all, they’ve never understood vulnerability. They’ve never experienced vulnerability. They can be taught it, they can read it in a book or a journal article, but they don't understand it.” [HCP participant]*

Building on this, another HCP participant emphasised the importance of providing tailored training to healthcare practitioners to ensure they have the suitable skills and understanding required to manage patients with additional support needs—and the positive impact this can have for the patients:

*“If you knew how to manage them [people experiencing homelessness] and you learned what was bad for them and so forth. If you were to bring more knowledge, you would bring more comfort to people” [HCP participant]*

#### 3.3.5 Peer mentoring and peer support

When re-imagining an inclusive model of dental care the possibility of creating community champions and dental peer mentors within the homeless community was discussed by participants. The first group identified several benefits of having peer mentors and peer support in dental teams in a new model of dental care and felt this would make them feel more comfortable attending the dentist if they were supported by someone that they knew:

*“What's good if you do go down the line about peer mentoring, is somebody that we know… would make us look forward to going to something like that.” [PwLE participant]*

In addition, participants highlighted that people with lived experience being trained as oral health peer mentors would be positively impacted on their level of education and improved chances of employment as well as extra motivated to make new changes to their current situation in the homeless system:

*“When you come off the streets, if you’ve got those certificates while being homeless, that shows a good character—somebody who wants to be motivated and wants to have a say and wants to make a difference in the position they are in.” [PwLE participant]*

Finally, participants identified as ‘community champions’ or dental peer mentors would act as a powerful point of reference to disseminate information amongst this population. Because each participant engages with multiple different services, providing training to these experts by experience to deliver oral health advice and signposting, would allow them to act as a link between services and service users helping people who don't engage with mainstream services to access this vital information.The recruitment of appropriate personnel for this role was highlighted as a key factor in making it successful by a healthcare practitioner in the second focus group:

*“Whether they are clinical champions or community champions, if you have someone who has lived it, gone through it, can empathise and relate. Then their success at bringing other people on that journey is going to be far better than somebody who has just read it in a book.” [HCP participant]*

## 4 Discussion

The barriers to accessing dental care while homeless identified by both groups are in line with the evidence from other studies (24–27). The comparability with these studies demonstrates that the social exclusion (2) and barriers to dental care experienced while homeless are a consequence of multiple different social, economic, cultural and systematic factors (28), and not the fault of the individual.

While the barriers to dental care are well established for the population, significant debate exists around the most effective way to deliver equitable dental care for patients experiencing homelessness (24, 25, 29, 30). In this study, healthcare professionals promoted the idea of an inclusive dental service, catering to the needs of multiple patient groups facing social exclusion, beyond people experiencing homelessness such as asylum seekers or individuals fleeing domestic abuse.

This study identified that the different stages of an individual's journey through homelessness were significant factors in a person's ability to engage with services and decide to make these positive changes. While factors around governance and recurring financial stability were identified as significant barriers to setting up an inter-sectoral dental service, multiple studies demonstrate the benefits of having some form of community outreach element to health promotion for these patients (31, 32). It is important to work with individuals experiencing homelessness to build their confidence and reduce the stigmatisation and othering that they are regularly subjected to.

Following the inclusion oral health framework (2), Paisi found a community-based homeless dental intervention to be a “highly successful, acceptable and accessible” model for dental care (33).

Hwang suggested that specific homeless services were effective at meeting the immediate needs of individuals, but that generic services integrated into healthcare systems deliver higher quality care (32). This implies that some form of integrated outreach service to address patients’ immediate needs can serve as a platform to build engagement and empower patients to see the benefit of making positive changes in the future. Daly confirmed this by highlighting that health services catering to the specific needs of homeless people provided a “safe environment which was in effect a comfort zone” and allowed them to establish “trusting networks and build their own social capital” (29). Trust and avoiding the need to repeat traumatic episodes to multiple different practitioners was an important theme discussed by participants with lived experience of homelessness, and it is important to consider how any prospective dental service would seek to build this between practitioners and patients.

This study reinforces the need to develop models for peer mentoring and peer support being included in dental services to facilitate patients’ engagement and help others overcome their barriers to accessing care by sharing their experiences. The study suggested these models could be based in community locations, with links to third-sector organisations, and look to establish strong working links with healthcare and statutory services. It was recognised thatin line with the inclusion oral health framework, further training should be delivered at undergraduate and postgraduate levels to ensure that the new generation of dental care professionals can understand the holistic needs of their patients and deliver trauma-informed care (34).

The benefits of training individuals, who have lived experiences of homelessness, to act as link workers between groups and services as dental peer mentors (35) was also a key finding in this study. However, participants recognised that as well as empowering individuals in the community to upskill (36) and take on additional responsibilities, peer mentors are already established within the community and connected to multiple different services. By utilising peer support in the capacity of link workers, homeless patients could have a bridge between healthcare services. Because missed appointments are a waste of vital resources (37), this model has been shown to improve the efficiency of clinical time, improve uptake of services and generally enhance sustainability and patient satisfaction for any service (33).

While sustainable alternative funding streams were identified as being a challenge to secure, the participants in the second focus group highlighted that establishing a service outside of the NHS remit would still be required to meet significant amounts of governance and regulation standards. This reinforces the need for third-sector organisations and healthcare providers to prioritise partnership working to meet the diverse needs of patients and service users. Effective frameworks for frictionless engagement between third-sector organisations and health providers should be considered to facilitate communication and information sharing between the sectors.

### 4.1 Future research

Qualitative and quantitative, peer-led research is required to identify the preferred method of service delivery for homeless patients. Consideration should be given to pilot studies that investigate the effectiveness of different models of care as well as the cost-benefit analysis of an intervention against the impact of missed dental appointments.

Further research is also needed on the barriers to inter-sectoral communication, to improve and streamline this to develop multi-disciplinary services. More research on peer mentoring, peer education and peer research would contribute to empowering individuals in the community and reduce the barriers to accessing dental care for others by acting as link workers between essential services.

### 4.2 Limitations of the study

Despite all the focus groups reached the number of participants usually recommended in the literature (between 6 and 8) the data was collected just post Covid-19 pandemic and lockdown. This increased the challenges to engage with homelessness services and recruit participants. Many services contacted and willing to support the study were unable to contribute as they were also reestablishing their work routines and communication with service users.

The potential influence of the principal investigator on the responses from research participants was mitigated by the strong long term trust build relationship formed with homelessness services that mediated the access to research participants. These local organisations guided the process of constant assessment and reassurance of participants being totally comfortable to express themselves.

In addition, this study covered one city in Scotland and did not reached a variety of homelessness participants and contexts such as those experiencing homelessness and living in rural areas of the country, those with severe mental health issues such as long term depression and anxiety that would not attend meetings in unfamiliar locations and in company of other participants they do not know. This may limit the findings being wider generalised.

While it is impossible to capture every view of adults experiencing homelessness, it would be beneficial to have more experts with experience to encapsulate a wider variety of inputs, including from third-sector managers and service providers.

## 5 Conclusion

While the current dental health system in Scotland is not fully tailored to respond to the holistic needs of patients experiencing homelessness, involving people with lived experience can be central to the process of reimagining new models of dental care focusing on reducing the health inequalities of vulnerable populations. These new models should be co-designed with people with lived experience and embedded in principles of trust, flexibility and peer mentoring. Practitioners taking a non-judgemental approach, aligned with a trauma-informed care environment and a well-trained workforce, would have more chances to effectively respond to the needs of people experiencing homelessness.

## Data Availability

On request, the thematic analysis data supporting the conclusions of this article will be made available by the authors, without undue reservation.
