# Supplementary material for: A stakeholder co-design approach to designing a dental service for adults experiencing homelessness
Source: Front Oral Health. 2024 Aug 26;5:1355429. doi: 10.3389/froh.2024.1355429 (PMC11381413; doi:10.3389/froh.2024.1355429)
Supplement: Supplementary file 1 [file Table1.docx]

**Tables:**

**Table 1 – Seven steps in the co-design framework for healthcare innovation. Adapted from M. Bird (17).**

| **Pre-design** | **Co-design** | **Post-design** |
| --- | --- | --- |
| **Step 1 – Contextual inquiry**   - Participant observation conducted in soup kitchens, homelessness services and on the streets that surrounded the area where key homelessness services in Glasgow were based/ immersion in the field. - Understanding the context of people experiencing homelessness regarding perceptions of oral health, needs and expectations. - Discussing the research question with managers and practitioners for health Boards and Third Sector, and with people with lived experience.   **Step 2 – Preparation / Planning for participation**   - Engagement and invitation of participants from both groups (people with lived experience of homelessness and health and social care practitioners). - PPI sessions - Obtaining consent from participants - Selection and knowledge exchange training of facilitators. - Identification of materials to support data collection. - Exchanging knowledge about the questions to guide the focus group. - Testing the instruments and making necessary change | **Stage 3** **– Framing the Issue**   - Focus groups (Group 1 and 2). - Presentation of generative design work. - Participants and facilitator engaged in critical reflection. - Appreciative inquiry   **Step 4 – Generative Design Ideas**   - Identification of latent needs and expectations about dental services - Knowledge exchange about actual dental services - Identification of challenges in accessing dental services - Starting a process of re-imagining / re-creating a new model of dental service provision   **Step 5 – Sharing Ideas**   - Identifying principles to base the foundation of this new model - Discussing what must change in actual services - Consolidation of the process of re-imagining / re-creating a new model of service delivery - Shared vision for the future | **Step 6 – Data Analysis**   - Preparation (transcription, analysis framework) - Organizing (coding) - Reporting findings   **Step 7 – Requirement translation**   - Action items - Feasible priorities to consider |

**Table 2: Themes, descriptive questions and prompts for Workshop 1 *(Experts-by-experience with lived experience of homelessness)***

| **Themes / Topics to be explored - Workshop 1** | **Broad Descriptive Questions** |
| --- | --- |
| **Knowledge / Awareness / Needs of Participants in Relation to Oral Health** | What do you understand is having a healthy mouth?  What behaviours or habits do you have that contribute to your oral health? (Good and bad)  Do you go to the dentist for check-up or only when in pain? |
| **Experiences trying to access a dentist in the last 18/24 months…** | Describe your experiences trying to access the dentist? *(Positive and Negative experiences)*  How did those experiences make you feel? |
| **Reactions / aspirations to different models of dental care used throughout the rest of the UK…** | Current model - Attend general practice?  Supported to attend general practice?  Designated service – Hospital/Community location? Emergency treatment only? |

**Table 3: Themes, descriptive questions and prompts for Workshop 2 *(Experts-by-experience with lived experience of homelessness and healthcare practitioners)***

| **Themes (Topics to be explored) – Workshop 2** | **Broad Descriptive Questions** |
| --- | --- |
| **Needs and contexts of people experiencing homelessness** | Introduce previous discussions from the first focus group around barriers and enablers…  Consider current service provision…In what ways does it meet the identified needs of patients? |
| **Service Components** | What should a high quality dental service for adults experiencing homelessness look like?  In an ideal world (blue sky thinking):  What should the core values of the service be?  What difficulties might arise trying to set up a service?  Realistic roadblocks to overcome? |
| **Service Design and Healthcare System Integration** | Given topics discussed today and expertise of participants, detail options for a prospective dental service for adults experiencing homelessness.  Relative to your field of expertise, how feasible are these options?  Can this dental service be linked into other homeless services? |

**Table 4: Participant’s characteristics for both focus groups**

| FOCUS GROUP 1 |  |  |
| --- | --- | --- |
| **People with lived experience (PwLE)** | **Gender** |  |
| Participant 1 | Male |  |
| Participant 2 | Female |  |
| Participant 3 | Male |  |
| Participant 4 | Male |  |
| Participant 5 | Female |  |
| Participant 6 | Female |  |
| Facilitator | Male |  |
|  |  |  |
| FOCUS GROUP 2 |  |  |
| **Health Care Provider (HCP)** | **Gender** | **Role** |
| Participant 1 | Male | General Dental Practitioner |
| Participant 2 | Female | Public Dental Service Clinician / Clinical Service Manager |
| Participant 3 | Male | Specialist in Dental Public Health |
| Participant 4 | Female | Oral Health Educator / Oral Health Improvement Manager |
| Participant 5 | Female | Operational Manager for Oral Health Improvement |
| Participant 6 | Male | Participant with Lived Experience of Homelessness |
| Facilitator | Male | Same Facilitator as other focus group / primary investigator |
